# Perspective: Hormones and immune development: bidirectional regulation between thyroid hormones and the immune system

**DOI:** 10.3389/fimmu.2026.1859721

**Published:** 2026-06-30

**Authors:** Weiling Zhang, Sitao Huang, Yingying Kang, Qingquan Zhao, Sixian Cheng, Zhengrong Zheng

**Affiliations:** Department of Thyroid Surgery, The Second Affiliated Hospital of Fujian Medical University, Quanzhou, China

**Keywords:** autoimmunity, endocrine–immune interactions, immune homeostasis, immunometabolism, reproductive immunology, T cell regulation, thyroid hormones

## Abstract

The immune system operates within a dynamic physiological environment shaped by systemic regulatory signals, among which endocrine factors are increasingly recognized as important modulators of immune function. Although thyroid hormones are well known for their roles in metabolism and development, their contribution to adaptive immunity, particularly in regulating T cell responses, remains incompletely understood. Emerging evidence suggests that thyroid–immune interactions are context-dependent across physiological and pathological conditions. In this Perspective, we propose that thyroid hormone signaling acts as a systemic regulator of T cell responses, integrating endocrine, immune, and metabolic cues to fine-tune immune activity. Mechanistically, this regulation involves thyroid hormone receptor–mediated pathways, interactions with cytokine networks, and immunometabolic programs that influence T cell differentiation and function. Clinically, thyroid dysfunction is associated with altered immune tolerance, autoimmune disease progression, and broader immune-related outcomes. Despite increasing interest in endocrine–immune crosstalk, current models remain fragmented. Future studies integrating longitudinal and multi-omics approaches are needed to better define thyroid–immune regulation and identify potential therapeutic targets.

## 1 Introduction

The immune system functions within a highly dynamic physiological environment and is continuously shaped by systemic regulatory signals beyond the classical immune network. Traditionally, immunological research has emphasized cell-intrinsic mechanisms and local microenvironmental interactions; however, growing evidence suggests that endocrine factors play an essential role in modulating immune responses at the organismal level (1). Hormonal networks provide context-dependent cues that influence immune cell development, differentiation, and effector function across a range of physiological states. Among these, thyroid hormones are well recognized for their central roles in metabolism and development (2–4), yet their contribution to adaptive immunity, including potential effects on T cell function, remains incompletely understood. This knowledge gap is especially evident under dynamic conditions such as pregnancy and inflammatory stress, where profound endocrine fluctuations coincide with significant immune adaptations (5, 6). Elucidating how thyroid hormone signaling interfaces with immune regulation in these contexts may uncover an underappreciated layer of systemic control over immune homeostasis and disease susceptibility.

Although emerging evidence has begun to highlight interactions between endocrine and immune systems, current conceptual models remain largely rooted in a static and compartmentalized view of immune regulation. Thyroid hormones have traditionally been studied in the context of basal metabolic control, with limited integration into frameworks of adaptive immune modulation. As a result, immune function is often interpreted independently of systemic endocrine fluctuations, an approach that may overlook critical regulatory layers. This limitation becomes particularly apparent when considering physiological transitions such as pregnancy and lactation, during which coordinated hormonal shifts are accompanied by profound changes in immune tolerance and T cell activity (5, 7). Similarly, in pathological conditions including autoimmune thyroid disorders and chronic inflammation, alterations in thyroid hormone signaling frequently coincide with dysregulated immune responses, yet their causal and mechanistic relationships remain poorly defined (3, 8). These observations suggest that conventional models, which treat endocrine and immune systems as parallel but largely independent entities, are insufficient to explain context-dependent immune behavior. A more integrative framework that incorporates dynamic endocrine signals into immune regulation is therefore needed.

## 2 Rethinking thyroid–immune interactions

In light of these limitations, a context-dependent thyroid–immune interaction framework can be proposed to better capture the dynamic interplay between endocrine signaling and adaptive immunity. Within this framework, thyroid hormones are not merely basal metabolic regulators but act as systemic modulators that calibrate T cell function according to physiological and environmental contexts. Under homeostatic conditions, thyroid hormone signaling contributes to the regulation of immune function, including T cell activation and cytokine production, and may influence the balance between effector and regulatory subsets (2, 4). Importantly, this regulatory effect is highly context-dependent. Different thyroid functional states may represent distinct immunological contexts with potentially divergent effects on T cell responses and immune homeostasis (4). Under euthyroid conditions, thyroid hormone signaling may contribute to the maintenance of baseline immune tone by supporting balanced T cell activation and cytokine production (4). In hypothyroid states, reduced thyroid hormone availability has been associated with impaired lymphocyte proliferation, decreased immune responsiveness, and a tendency toward hyporesponsive or regulatory immune profiles (9). In contrast, hyperthyroidism is frequently linked to enhanced inflammatory activity, increased cytokine production, and expansion of effector T cell responses, potentially favoring autoimmune activation (8, 10). These observations suggest that the immunological consequences of thyroid hormone signaling may vary not only in magnitude but also in functional direction depending on the endocrine context. During physiological transitions such as pregnancy, coordinated changes in immune tolerance and T cell activity are well documented (5), These processes occur alongside significant fluctuations in thyroid hormone levels, suggesting a potential role for endocrine signals in dynamically modulating immune responses in response to systemic demands. In pathological conditions, including chronic inflammation and autoimmune disorders, alterations in thyroid hormone signaling are frequently associated with immune imbalance, particularly in T cell-mediated responses (4). Collectively, these observations support a model in which thyroid hormone signaling functions as a context-dependent regulator of adaptive immunity. The underlying mechanisms likely involve coordinated regulation of immune signaling, cytokine networks, and downstream cellular programs, which together fine-tune T cell responses across diverse physiological and pathological states.

## 3 Mechanistic basis of thyroid hormone–mediated T cell modulation

Mechanistically, the regulatory effects of thyroid hormones on T cell function are mediated through both genomic and non-genomic pathways, primarily involving thyroid hormone receptors (TRα and TRβ), which are expressed in immune cells (2, 4). Upon ligand binding, these receptors act as transcriptional regulators that modulate gene expression related to immune cell activation and proliferation (11). In addition, non-genomic signaling pathways, including PI3K/Akt and MAPK cascades, have been implicated in mediating rapid cellular responses to thyroid hormones (12). In addition, thyroid-stimulating hormone and thyroid hormone signaling have been shown to directly influence immune cell activation and cytokine release, linking endocrine signals to inflammatory responses through receptor-mediated intracellular pathways (4).

At the cellular level, thyroid hormone signaling has been implicated in shaping immune cell differentiation and function, although direct evidence in specific T cell subsets remains limited (2, 4). In adaptive immunity, the balance between pro-inflammatory Th17 cells and immunosuppressive regulatory T cells (Tregs) is a key determinant of immune responses. This balance is regulated by well-characterized pathways, including IL-6/STAT3 signaling, which promotes Th17 differentiation, and transcriptional regulation of Foxp3, which governs Treg stability and function (13, 14).

Beyond receptor-mediated and transcriptional control, emerging evidence highlights a critical role of immunometabolic reprogramming in linking thyroid hormone signaling to T cell fate. Notably, recent multi-omics analyses in pregnant women with hypothyroidism have revealed activation of sphingolipid metabolism, with increased sphingosine levels closely associated with elevated Th17/Treg ratios (15). These metabolic alterations were further correlated with increased IL-6 levels, suggesting that sphingolipid-mediated inflammatory signaling may drive Th17 polarization and immune imbalance (15). Consistent with this metabolic–immune coupling, key metabolic pathways, including glycolysis and lipid metabolism, have been shown to govern T cell activation and lineage specification, with metabolic reprogramming acting as a determinant of effector versus regulatory T cell fate decisions (16, 17). Distinct T cell subsets are characterized by specific metabolic programs that actively shape their differentiation and functional stability (18, 19). Tregs primarily rely on oxidative phosphorylation (OXPHOS) and fatty acid oxidation to sustain their suppressive activity, whereas pro-inflammatory Th17 cells preferentially utilize aerobic glycolysis to support rapid proliferation and cytokine production (18). Importantly, these metabolic states are not merely secondary consequences of lineage commitment but function as active regulators of T cell fate decisions (17). In this context, thyroid hormone signaling may contribute to immune polarization by modulating cellular metabolic pathways, thereby influencing the balance between inflammatory and regulatory T cell responses across different physiological and pathological conditions. Collectively, these findings support a multi-layered regulatory model in which thyroid hormone signaling integrates receptor-mediated pathways, cytokine networks, and metabolic circuits to orchestrate T cell responses in a context-dependent manner.

## 4 Clinical relevance of thyroid–immune crosstalk

The context-dependent regulatory role of thyroid hormones in T cell function carries important clinical implications across multiple physiological and pathological settings. In reproductive immunology, maternal–fetal tolerance is established through tightly regulated immune adaptations during pregnancy, involving coordinated changes in both innate and adaptive immune responses (20, 21). Disruption of this finely balanced immune state has been associated with adverse pregnancy outcomes, including miscarriage and preterm birth. Thyroid dysfunction has also been consistently linked to unfavorable pregnancy outcomes and systemic physiological alterations (22, 23), suggesting a potential interaction between endocrine status and immune regulation during gestation.

In autoimmune conditions, particularly Graves’ disease and Hashimoto’s thyroiditis, thyroid hormone dysregulation is closely associated with aberrant T cell responses and chronic inflammation, contributing to disease onset and progression (8, 10). Clinical studies further demonstrate altered immune profiles, including imbalances in T cell subsets and increased inflammatory activity, in patients with thyroid dysfunction (24, 25), supporting a functional link between endocrine dysregulation and immune-mediated pathology.

Beyond these conditions, systemic endocrine disturbances have been associated with altered immune responsiveness in chronic inflammatory diseases and cancer. Thyroid dysfunction has been linked to elevated inflammatory markers and adverse clinical outcomes, suggesting that endocrine imbalance may influence disease progression through immune-mediated mechanisms (15). In oncology, where T cell function is central to anti-tumor immunity, metabolic and hormonal states have emerged as important determinants of treatment response and immune efficacy. Collectively, these clinical observations highlight the relevance of thyroid hormone, mediated endocrine, immune interactions in shaping disease susceptibility, progression, and therapeutic outcomes.

## 5 Challenges and future directions

Recent advances in multi-omics technologies provide unprecedented opportunities to dissect endocrine–immune interactions at high resolution; however, several critical challenges remain in translating these findings into a coherent mechanistic and clinical framework. At the experimental level, most current studies rely on static measurements of hormone levels or immune cell profiles, which fail to capture the dynamic and context-dependent nature of endocrine–immune crosstalk. In particular, temporal fluctuations in thyroid hormone signaling during physiological transitions such as pregnancy or inflammatory stress are rarely integrated with longitudinal immune profiling, limiting the ability to infer causality.

At the mechanistic level, distinguishing direct receptor-mediated effects from downstream metabolic and inflammatory pathways remains a major challenge. While emerging studies highlight the role of immunometabolic reprogramming, the relative contribution of specific pathways—such as cytokine signaling versus metabolic remodeling—in shaping T cell fate has not been systematically defined. In addition, current omics-based approaches, including transcriptomics and metabolomics, often lack sufficient resolution to link endocrine signals to specific immune cell subsets in a context-dependent manner, and inconsistencies across analytical platforms further complicate interpretation.

From a clinical perspective, although associations between thyroid dysfunction and immune-related outcomes have been widely reported, the identification of actionable biomarkers and therapeutic targets remains limited. A key challenge lies in distinguishing causal regulatory mechanisms from secondary systemic changes, as well as translating complex endocrine–immune interactions into clinically applicable indicators. Addressing these challenges will require integrative strategies that combine longitudinal endocrine–immune profiling, high-resolution multi-omics approaches, and functional validation in relevant disease models. Such efforts are expected to advance the field toward a more precise and mechanistically grounded understanding of thyroid–immune regulation.
